# CurT dosage quantitatively affects thylakoid structure and PSII performance in *Synechocystis* sp. PCC 6803

**DOI:** 10.1111/tpj.71132

**Published:** 2026-09-21

**Authors:** Matthias Ostermeier, Anne‐Christin Pohland, Patrick Reinmöller, Marcel Dann

**Affiliations:** ^1^ Plant Molecular Biology, Faculty of Biology Ludwig‐Maximilians University Munich Großhaderner Straße 2‐4 Planegg‐Martinsried 82152 Germany; ^2^ Bio‐Inspired Energy Conversion Technical University of Darmstadt Schnittspahnstraße 10 Darmstadt 64287 Germany

**Keywords:** CurT, thylakoids, thylapse, convergence zone, *Synechocystis*, cyanobacteria

## Abstract

The photosynthetic thylakoid membrane systems of many oxygenic photosynthesizers form elaborate three‐dimensional structures. Curvature Thylakoid (CurT/CURT1) proteins are central regulators of thylakoid architecture in cyanobacteria and plants, driving both grana stacking and the formation of thylakoid convergence zones (TCZs) by promoting membrane curvature while also contributing to green‐algal chloroplast and cyanobacterial cell division. While the functional role of grana stacks in terrestrial photosynthesis is well established, the physiological significance of cyanobacterial TCZs remains unclear. Here, we investigate the physiological role of TCZs and thylakoid ultrastructure in *Synechocystis* sp. PCC 6803 by quantitatively assessing how CurT protein abundance shapes thylakoid membrane organization, TCZ frequency, and thylakoid layering, and how these architectural features relate to photosynthetic efficiency and growth in a set of *curT* expression mutants. By correlating cellular CurT levels with thylakoid structural and physiological parameters of the system, we define a quantitative relationship between CurT abundance, thylakoid architecture, photosystem II (PSII) performance, and growth. Our results reveal a non‐linear threshold‐like relationship best captured through a logarithmic regression model: minimal CurT suffices to restore TCZ formation, partially recovering PSII activity and growth rate, while CurT overexpression further enhances late‐stage biomass accumulation through an apparently PSII‐activity independent mechanism. Together, these findings identify CurT as a dose‐dependent key determinant of thylakoid structure and underscore the functional importance of thylakoid membrane architecture for efficient photosynthesis and growth.

## Significance Statement

We provide evidence for CurT acting as a dosage‐dependent key regulator of cyanobacterial membrane organization and for the physiological significance of CurT‐induced thylakoid ultrastructure in the model cyanobacterium *Synechocystis* sp. PCC 6803. Furthermore, changes in PSII assembly and photoautotrophic growth render CurT a potential target for improving cyanobacterial light‐energy capture and conversion.

## INTRODUCTION

Cyanobacteria are one of the most ancient and taxonomically diverse bacterial clades defined by their capacity for oxygenic photosynthesis (Garcia‐Pichel et al., 2020), relying on the tandem operation of two light‐driven membrane‐embedded multi‐protein redox enzyme complexes, that is, Photosystem II (PSII) and Photosystem I (PSI), to transfer electrons from water to ferredoxin *via* several intermediate electron carriers (Komenda et al., 2024; Qi et al., 2023).

The overall architecture of protein complexes driving the photosynthetic electron transport chain is highly conserved across cyanobacteria and is shared, with only minor modifications, by chloroplasts of eukaryotic algae and plants (Nickelsen & Rengstl, 2013). Apart from early‐branching *Gloeobacterales* (Koyama et al., 2008; Rexroth et al., 2011), all these complexes are physically embedded in a specialized internal membrane system known as thylakoid membranes (TMs) (Ostermeier et al., 2024). Despite their conserved function, thylakoid systems show striking architectural diversity among oxygenic photosynthesizers (Mareš et al., 2019; Ostermeier et al., 2024). In land plant chloroplasts, TMs are commonly differentiated into loose stroma lamellae and tightly appressed and contorted grana stacks (Austin & Staehelin, 2011), which are considered an evolutionary adaptation to dry, high‐light terrestrial environments (Gu et al., 2022). Meanwhile, cyanobacterial thylakoids are frequently arranged as concentric lamellae, parallel stacks, or irregular networks that pervade the cytoplasm, optimizing light capture and electron transport in low‐light aquatic environments (Mareš et al., 2019). The precise architecture of these membrane systems varies markedly among species, reflecting adaptations to distinct ecological niches and cell morphologies.

*Synechocystis* sp. PCC 6803 (hereafter *Synechocystis*), a widely studied model organism, provides a prime example of specialized thylakoid architecture. In *Synechocystis*, TMs are organized into discrete peripheral layers or fascicles that lie just beneath the cytoplasmic membrane, leaving a central cytoplasmic core largely devoid of photosynthetic membranes (Heinz et al., 2016; Liberton et al., 2006; Ostermeier et al., 2022, 2024; Van De Meene et al., 2006). Within this peripheral zone, thylakoid convergence zones (TCZs) appear at specific sites where TMs curve toward the plasma membrane (PM) into a compact junction before branching again into individual stacks and forming thylapses – in analogy to neural synapses – as close contact sites that likely facilitate lipid and small molecule exchange (Rast et al., 2019). This process may be aided by Vesicle‐inducing protein in plastids 1 (VIPP1) (*aka* inner membrane‐associated protein of 30 kDa IM30)‐like proteins whose lipid‐trafficking function is conserved even in *Gloeobacter* (Ma et al., 2025). The geometry of *Synechocystis* thylapses at the tip of the TCZ region is often triangular or trapezoidal in cross‐section, creating localized microdomains distinct from the more planar thylakoid lamellae (Rast et al., 2019). Recent studies have shown that two key membrane‐shaping factors, CurT and AncM (anchor of convergence membranes), act together to generate and stabilize TCZs in *Synechocystis* (Ostermeier et al., 2022). Here, CurT is the cyanobacterial homolog of CURVATURE THYLAKOID1 (CURT1) proteins first characterized in *Arabidopsis thaliana* (hereafter *Arabidopsis*) where analysis of loss‐ and gain‐of‐function mutants demonstrated that CURT1 proteins oligomerize at grana margins and induce the extreme membrane curvature required for grana stacking (Armbruster et al., 2013; Pribil et al., 2018). Recombinant *Arabidopsis* CURT1 protein was shown to suffice to tubulate liposomes *in vitro*, rendering membrane bending an intrinsic, CURT1‐mediated phenomenon (Armbruster et al., 2013). Grana margins in plant chloroplasts are highly curved thylakoid subdomains enriched in factors involved in photosystem biogenesis and repair (Pribil et al., 2018). In the absence of CURT1, chloroplasts form flat, lobe‐like thylakoids with reduced grana margins and impaired photosynthetic performance. Conversely, CURT1 overexpression produces slimmer, higher grana with more membrane layers and a reduced grana diameter compared with WT plants (Armbruster et al., 2013, Pribil et al., 2018).

In cyanobacteria, evidence points toward CurT fulfilling an analogous role. *Synechocystis* CurT tubulates liposomes *in vitro* (Heinz et al., 2016), can be functionally substituted by *Arabidopsis* CURT1A (Armbruster et al., 2013), and, while localizing broadly across TMs, accumulates at TCZs (Heinz et al., 2016). CurT‐deficient *Synechocystis* mutants completely lack organized TCZs, and their thylakoid layers instead form disordered rings or crumpled arrays. This structural disruption coincides with a ~50% reduction in PSII assembly under photomixotrophic conditions, likely due to PSII assembly and repair machinery being concentrated in TCZs (Heinz et al., 2016) akin to chloroplast grana margins (Ostermeier et al., 2025; Pribil et al., 2018). Live‐cell imaging further showed that CurT forms tubular networks at the periphery of the thylakoid system, consistent with a role in shaping membrane curvature at nascent convergence sites (Heinz et al., 2016; Ostermeier et al., 2022). Recently, in *Synechococcus elongatus* PCC 7942, CurT was shown to be also responsible for thylakoid architecture and photosynthetic performance (Zhang et al., 2025), thus corroborating a phylogenetically conserved role of CurT/CURT1‐like proteins.

The AncM protein has been identified through a suppressor screen in a *curT*‐deficient *Synechocystis* mutant (Ostermeier et al., 2022). This single‐pass transmembrane protein localizes to punctate sites near the cell envelope, anchoring thylapse regions to the plasma membrane precisely where CurT‐induced thylakoid curvature is most pronounced. In the absence of AncM, TCZs lose contact with the plasma membrane, resulting in enlarged lamellar stacks and diminished functional integrity (Ostermeier et al., 2022). Thus, CurT has been suggested to provide the membrane‐bending force shaping the thylakoid lamellae, while AncM is hypothesized to secure curved membrane sectors in place, presumably forming a funnel‐like structure toward the PM that defines PSII assembly microdomains at thylapse regions (Ostermeier et al., 2022, 2025; Rast et al., 2019).

Structurally, CurT is assumed to oligomerize into high‐molecular‐mass complexes that insert into the outer leaflet of the thylakoid bilayer, imposing local curvature that both concentrates and excludes specific lipid species (Heinz et al., 2016). The resulting curvature may promote spatial segregation of PSII assembly intermediates and create curvature‐sensing niches that recruit additional biogenesis factors (Ostermeier et al., 2025; Rengstl et al., 2011). A sufficient degree of membrane curvature in turn could be essential for optimal phycobilisome‐photosystem interaction and may also influence the contact of other soluble proteins with membrane‐bound components (Lea‐Smith et al., 2016). The precise molecular mechanisms and the extent to which such microdomain formation depends on protein–protein interactions, induced changes in lipid environments, and synergistic effects thereof, however, remain to be elucidated.

Previous studies have established that CurT deficiency affects both cell division and TCZ formation in cyanobacteria (Dann et al., 2025; Heinz et al., 2016; Ostermeier et al., 2022). Significant progress has also been made in uncovering the molecular foundation of TCZ and thylapse formation (Huokko et al., 2021; Ostermeier et al., 2024). In addition, TCZs have been implicated in PSII assembly (Ostermeier et al., 2025). However, the apparent absence of TCZ‐like structures in many cyanobacterial taxa raises questions about their physiological relevance in *Synechocystis*. Moreover, the quantitative effects of CurT depletion and overproduction on photosynthesis remain uncharacterized. To address this, we analyzed a set of recently established *curT* expression‐level mutants (Dann et al., 2025) with elevated (overexpression, OE), reduced (complementation strain, compl.), and abolished (knock‐out, KO) cellular CurT levels, as well as the parental wildtype (WT). We examined how relative CurT abundance influences thylakoid structural features measured as average numbers of TCZs and thylakoid layers per fascicle per cell, as well as photosynthetic efficiency assessed by PSII dark and light‐adapted quantum yield and net‐oxygen evolution rates under photoautotrophic growth conditions. We found evidence of CurT depletion and enrichment having opposite effects on growth and thylakoid organization: OE cells outperform WT, whereas compl. cells display intermediate phenotypes between KO and WT. Notably, the compl. strain revealed a non‐linear relationship between CurT levels, TCZ formation, and PSII performance, indicating that even minimal amounts of cellular CurT are sufficient to support TCZ formation and growth, while overexpression of CurT results in improved growth and quantum yield metrics without improving PSII enzymatic activity.

## RESULTS AND DISCUSSION

### TCZ formation in *Synechocystis* quantitatively depends on CurT

To study the quantitative effects of cellular CurT protein level modulation in *Synechocystis* on thylakoid ultrastructure, PSII performance, and growth as an integrating parameter for photosynthetic yield, a set of previously established *curT* expression‐level mutants (Dann et al., 2025) was initially subjected to TEM analysis, with cellular cross‐sections being analyzed as two‐dimensional proxies for total three‐dimensional cellular abundance of thylakoid‐system structural features. The *curT* expression‐level mutant set comprises a parental wildtype (WT), overexpression (WT + *slr0168*::*curT*_SpecR), knock‐out (Δ*curT*_SpecR knock‐out mutant, KO) and complementation (compl.) strain (Δ*curT*_SpecR *slr0168*::*curT*_CmR), the latter of which was previously found to accumulate only trace amounts of cellular CurT (Dann et al., 2025). Of note, the compl. strain was obtained by transforming incompletely segregated *curT* KO mutant cells with the complementation construct prior to complete segregation of the chromosomal KO allele (Dann et al., 2025) as segregated *curT* KO mutants were found to lose their natural competence as previously reported (Heinz et al., 2016). Cells were cultivated under photoautotrophic conditions at 25 °C and 50 μmol m^−2^ s^−1^ of warm‐white LED light in BG11 medium until the mid‐exponential growth phase was reached (see Methods).

**Figure 1:**
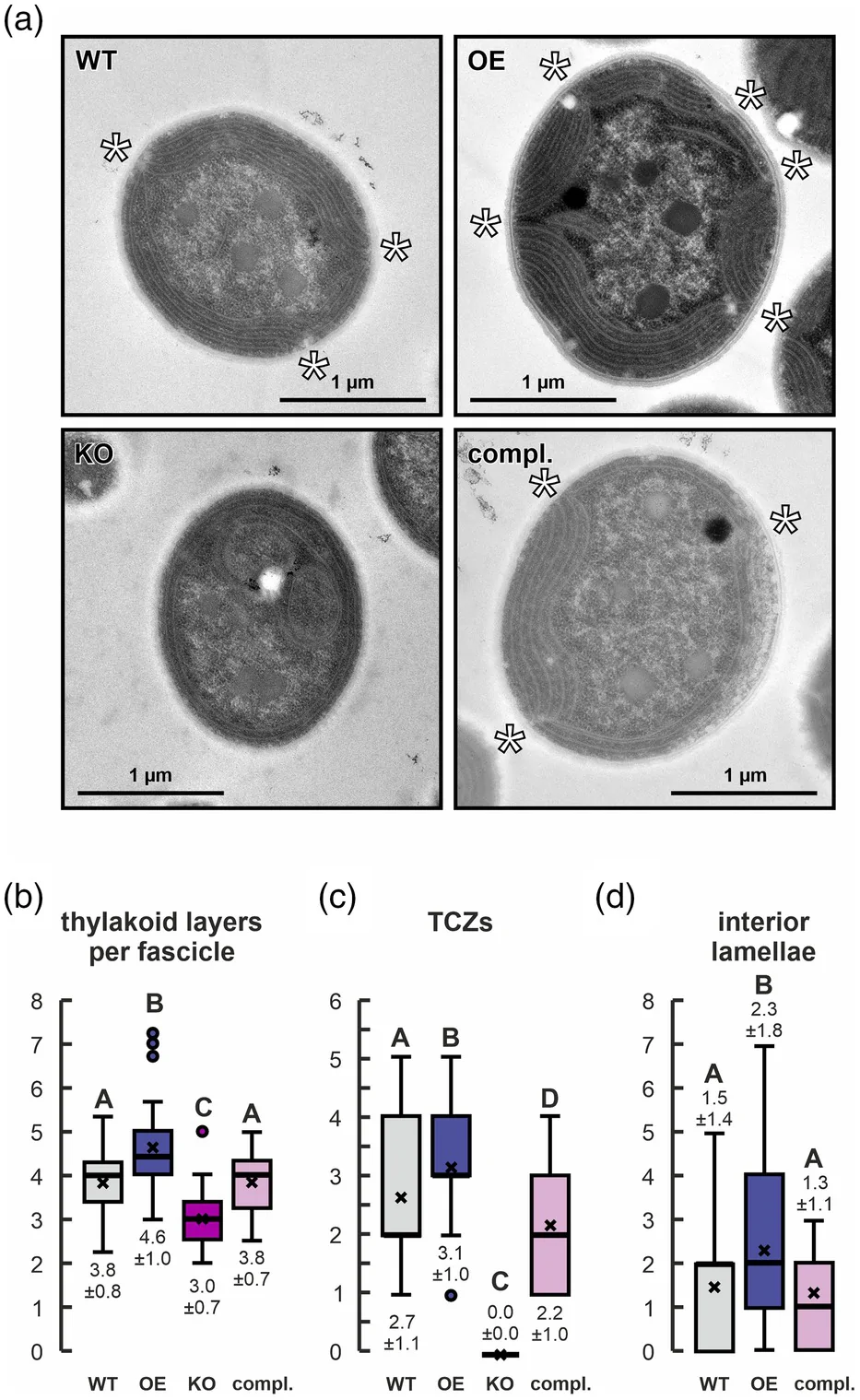
Dosage effects of cellular CurT levels on thylakoid‐system structure. (a) Representative transmission electron micrographs of *Synechocystis curT* expression‐level mutant cell thin sections. WT, wildtype; OE, *curT* overexpression; KO, *curT* knock‐out; compl., *curT* complementation strain. White asterisks indicate thylakoid layers grouped to peripheral fascicle. Scale bars (1 μm) are indicated. (b) Average number of thylakoid layers per peripheral fascicle (i.e., thylakoid‐system segments interrupted by TCZs, see white asterisks in (a)) in mid‐exponential growth phase *Synechocystis curT* expression‐level mutants. Note that in case of the KO mutant in absence of TCZs, this measure indicates the number of thylakoid layers per cell. (c) Number of thylakoid convergence zones in mid‐exponential growth phase *Synechocystis curT* expression‐level mutants. (d) Number of thylakoid lamellae protruding into the cell center of mid‐exponential growth phase *Synechocystis curT* expression‐level mutants. In panels (b‐d), boxplots represent data of *n* = 37/38/37/35 individual cells for WT/OE/KO/compl., respectively. Estimates are derived from one thin section per observed cell. Boxplots center lines indicate medians, crosses indicate averages, boxes indicate the 25th–75th percentiles, whiskers indicate the 1.5‐fold interquartile range, and circles indicate outliers. Values below plots indicate averages ± standard deviation. Uppercase letters indicate statistically significant differences (*P* < 0.05) according to *post hoc* Bonferroni–Holm‐corrected Tukey HSD after significant among‐group differences (*P* < 0.05) had been determined by two‐sided one‐way ANOVA.

TEM‐micrographs of photoautotrophically grown *Synechocystis curT* expression‐level mutant cell thin sections (Figure 1a, Figure S1) revealed significant architectural differences among strains. OE cell thylakoid fascicles contained +21% thylakoid sheets on average (*P* < 0.001), whereas the number of KO thylakoid membrane layers showed a significant decrease as compared to WT (−21%; *P* < 0.001) (Figure 1b). Meanwhile, the compl. strain did not show any obvious difference to WT (+0.1%; *P* = 0.920) (Figure 1b). The same cells showed a significant increase in the average number of TCZs in OE (+16%; *P* = 0.031), while KO cells completely lacked (−100%; *P* < 0.001) and compl. cells displayed a moderate yet statistically significant decrease in average numbers of TCZs per cell (−19%; *P* = 0.024), respectively (Figure 1c). These results indicate a quantitative effect of cellular CurT levels on both TCZ formation and thylakoid sheet number, complementing earlier qualitative observations on CurT being essential for TCZs formation (Heinz et al., 2016). Notably, this relationship appears to be non‐linear as even trace amounts of cellular CurT in the compl. strain enable TCZ formation at approximately 79% the rate of WT cells (see Figure 1c). This suggests that while CurT is essential for TCZ formation, most cellular CurT in WT is likely involved in other cellular processes or exists in an inactive protein reservoir. Additionally, OE cells displayed a significant increase in the average number of thylakoid lamellae protruding into the cell interior as compared to WT (+57%; *P* = 0.031), while compl. cells showed a slight, non‐significant reduction (−9%; *P* = 0.707) (Figure 1d). Such protruding lamellae may enhance the efficiency of targeting RNA‐binding proteins (RBPs) to TMs oriented toward the cell interior, possibly enabling the capture of, for example, *psbA* mRNA (Mahbub et al., 2020), its transport and insertion of the D1 polypeptide, thus presumably facilitating PSII biogenesis at the TCZs (Ostermeier et al., 2025). In KO cells, no such lamellae could be observed, while vesicle‐shaped thylakoid segments occupied the inside of most cells, consistent with previous findings.

### Cellular CurT levels affect PSII performance parameters under photoautotrophic growth conditions

After transmission electron microscopy had revealed a titration effect of expected CurT levels on cellular ultrastructure, culture samples of CurT expression‐level mutants were analyzed by whole‐cell absorbance spectrometry, in‐gel fluorescence assays, and immunoblotting to assess combined allophycocyanin + phycocyanin (APC + PC), CurT, PsbA (D1), PsbB (CP47), AtpB, and PsaA protein levels, and by methanolic pigment extraction, oxygen evolution, and chlorophyll fluorescence measurements to correlate thylakoid morphology, growth, and photosynthetic performance with cellular CurT levels.

**Figure 2:**
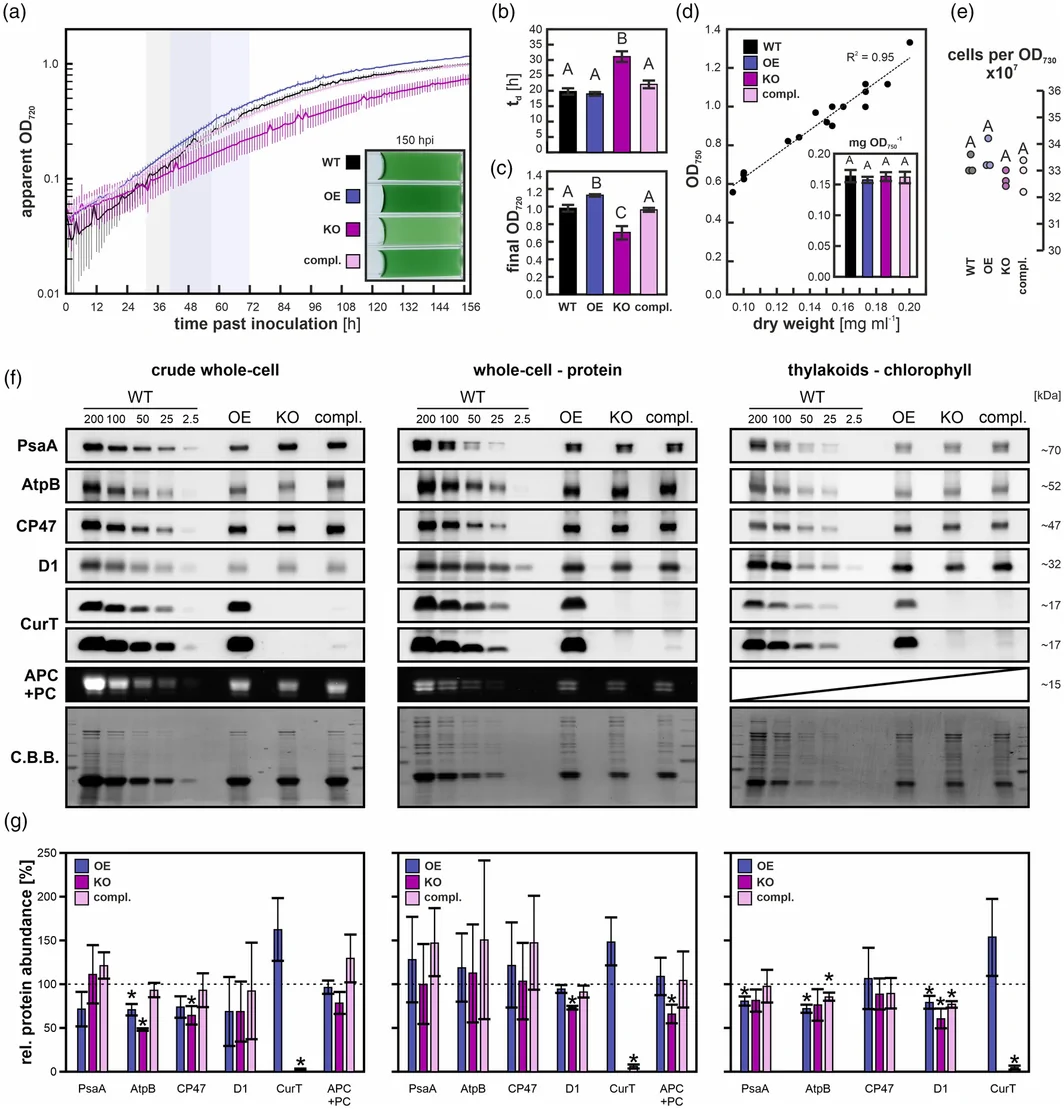
Photoautotrophic growth, CurT, and PSII marker protein level quantification in *Synechocystis curT* expression‐level mutants. (a) Growth curves of *curT* expression‐level mutants at 25 °C and 50 μmol photons m^−2^ s^−1^, monitored as apparent OD_720_ (Multicultivator inbuilt photometer; see Methods). Curves represent averages; error bars represent standard deviations of *n* = 3 biological replicates used for subsequent physiological characterization. Inset shows representative culture phenotypes at 150 h past inoculation. WT, wildtype; OE, *curT* overexpression; KO, *curT* knock‐out; compl., *curT* complementation strain. Shaded areas indicate time windows for calculation of exponential growth phase duplication times (gray, WT/OE/compl.; blue, KO). (b) Exponential growth phase duplication times t_d_. Columns show averages; error bars indicate standard deviations of *n* = 3 biological replicates. (c) Final apparent OD_720_ at 150 h past inoculation. Columns show averages; error bars indicate standard deviations of *n* = 3 biological replicates. (d) Linear correlation between exponential‐phase culture OD_750_ and dry weight of cell material. Inset shows corresponding dry‐weight‐to‐unit‐OD_750_ ratios. Columns show averages; error bars indicate standard deviations of *n* = 4 biological replicates. (e) Number of cells per unit OD_730_ as estimated by Neumann chamber count. Individual data points for *n* = 3 biological replicates are shown. (f) Protein levels as assessed by immunoblot analysis of crude whole‐cell protein extracts (left), protein‐adjusted cell homogenate (30 μg total protein per lane; middle), and chlorophyll‐adjusted thylakoid preparation (5 μg Chl *a* per lane; right) obtained from mid‐exponential‐phase cultures 3–4 days past inoculation. Combined allophycocyanin and phycocyanin (APC + PC) levels were assessed by in‐gel fluorescent assay prior to blotting of SDS‐PA gels (not assessed for thylakoid membrane preparations due to water‐solubility). PVDF membrane Coomassie Brilliant Blue (C.B.B.) staining served as loading control. Approximate molecular weight markers are indicated. (g) Enhanced chemiluminescence and fluorescent signals were quantified to determine protein levels relative to 100% WT samples. Data shown represents *n* = 3 biological replicates. Columns show averages; error bars indicate standard deviations. Calibration curves are provided in Figure S2. Uppercase letters indicate statistically significant differences (*P* < 0.05) according to *post hoc* Bonferroni–Holm‐corrected Tukey HSD after significant among‐group differences (*P* < 0.05) had been determined by two‐sided one‐way ANOVA. Asterisks indicate statistically significant differences from the WT control (*P* < 0.05) according to two‐sided Student's *t*‐test.

Under photoautotrophic growth conditions, absence of CurT led to slower growth of KO cells, with average duplication times increasing by 57% (*P* < 0.001), while CurT depletion in the compl. strain showed a modest, non‐significant increase in average duplication time (+12%, *P* = 0.100) as compared to WT (Figure 2a,b). In contrast, OE cells, displayed slightly shorter average duplication time (−4%; *P* = 0.458; Figure 2a,b), but a significantly increased average OD_720_ at the end of the growth experiment (i.e., after 152 h of growth; +15%, *P* = 0.007; Figure 2c). These results were in line with previous observations under photomixotrophic growth conditions (Dann et al., 2025; Heinz et al., 2016). Exponential‐phase culture OD_750_ was found to closely correlate in a linear fashion with cellular dry weight (R^2^ = 0.95; Figure 2d), and cell count per unit OD_730_ was found virtually identical among all mutant strains (OE +1%; KO −2%; compl. −1%; Figure 2e). This finding corroborates previous reports on close correspondence between cell number and OD_750_ in parental *Synechocystis* WT and *curT* knock‐out mutants (3.9 × 10^7^ and 3.7 × 10^7^ cells ml^−1^ OD_750_^−1^) (Heinz et al., 2016), and *Synechocystis* OD_730/750_ and biomass in general (Myers et al., 2013), rendering OD‐normalization in principle suitable for cell‐number adjustment in subsequent immunoblot analyses and physiological measurements. As *curT* mutants grown photomixotrophically have been reported to display reduced cellular chlorophyll content (Heinz et al., 2016), comparative immunoblot analyses were performed on whole‐cell protein extracts obtained from OD_750_‐adjusted intact cells directly lysed in 2x lysis buffer (‘crude whole‐cell’), protein‐adjusted whole‐cell homogenate (‘whole‐cell protein’), as well as Chl *a*‐adjusted thylakoid membrane preparations to ensure hydrophobic membrane protein recovery (Komenda et al., 2004).

Immunoblotting confirmed the absence of CurT in KO cells across all sample preparations, while compl. cells displayed an average decrease by 95–97% and OE cells displayed an average increase by 47–62% relative to WT, respectively (Figure 2f,g), corroborating results from mixotrophic cultures (Dann et al., 2025). Importantly, low‐level CurT detection was found robust in crude whole‐cell protein extract, while D1, CP47, and AtpB protein recovery by this method were found inconsistent (D1) and sub‐stoichiometric (CP47, AtpB) in comparison to protein‐adjusted cell homogenates and chlorophyll‐adjusted thylakoid preparation (Figure 2f,g). This renders protein‐adjusted whole‐cell homogenate and chlorophyll‐adjusted TM preparations more reliable bases for quantitative comparison of large and/or hydrophobic marker proteins. As no major differences were observed for CurT level estimates among all three protein extraction protocols, quantitative CurT protein level estimates derived from OD‐adjusted crude whole‐cell protein extracts were used for subsequent correlation analyses (see Figure 4).

**Figure 3:**
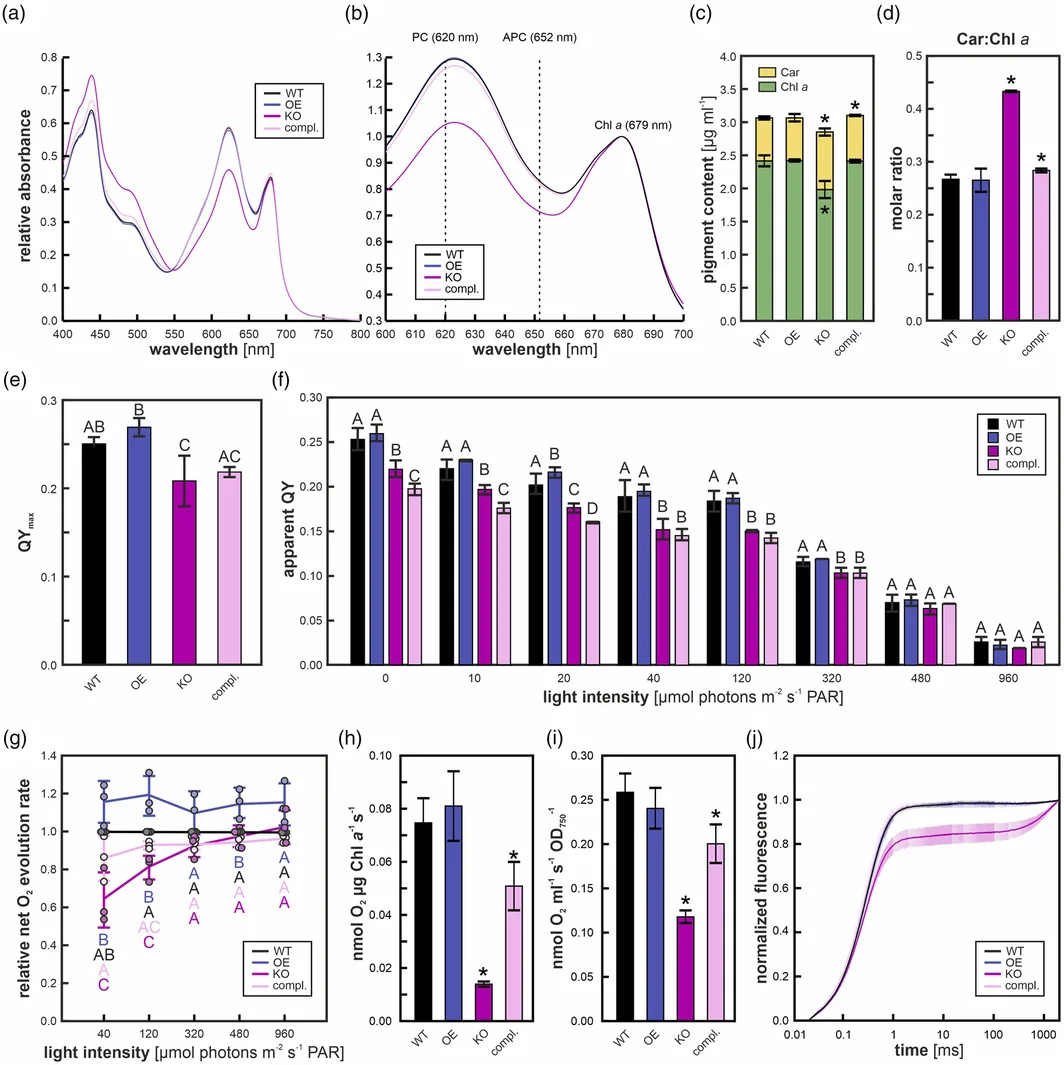
Cellular CurT protein levels affect pigmentation and PSII performance parameters in *Synechocystis*. (a) UV/Vis absorbance spectra of intact *Synechocystis* cells. Traces represent average absorbance spectra of *n* = 3–4 biological replicates. WT, wildtype; OE, *curT* overexpression; KO, *curT* knock‐out; compl., *curT* complementation strain. (b) Whole‐cell absorbance spectra shown in panel (a) normalized to 679 nm (Chl *a*) reveal relative depletion of phycobilisomes in the KO strain. (c) Chl *a* and carotenoid (Car) contents of OD_750_ = 0.75 cell equivalents for *n =* 4 biological replicates of mid‐exponential growth phase *Synechocystis curT* expression‐level mutant cultures (methanolic pigment extract). (d) Molar ratios of total carotenoids to Chl *a* (Car:Chl_*a*_) inferred from data shown in panel (c). (e) PSII maximum quantum yield (QY_max_; +10 μM DCMU) of mid‐exponential growth phase dark‐adapted *Synechocystis curT* expression‐level mutants for *n =* 4–6 biological replicates. (f) Light curve of apparent QY in mid‐exponential growth phase *Synechocystis curT* expression‐level mutants for *n =* 3 biological replicates. (g) Light‐response curve of net‐oxygen (O_2_) evolution rates per unit OD_750_ relative to WT measured in light‐adapted mid‐exponential growth phase *Synechocystis curT* expression‐level mutants at 25 °C for *n =* 3 biological replicates. (h, i) PSII activity determined as steady‐state oxygen evolution in presence of 0.5 mM DCBQ and 1 mM K_3_Fe(CN)_6_ under saturating light conditions (500 μmol photons m^−2^ s^−1^) at 25 °C. Measurements were performed on *n =* 4 biological replicates of undiluted culture samples grown for 3 days (mid‐exponential growth phase). Data was normalized to OD_750=_1 (h) and Chl *a* content (i), respectively. (j) OJIP transients in presence of 10 μM DCMU recorded after 10 min dark incubation. Curves and shaded areas represent averages ± standard deviations of *n* = 6/3/4/3 biological replicates for WT/OE/KO/compl, respectively. In panels (c–f/g,h), columns show averages; error bars indicate standard deviations of biological replicates. Uppercase letters indicate statistically significant differences (*P* < 0.05) according to *post hoc* Bonferroni–Holm‐corrected Tukey HSD after significant among‐group differences (*P* < 0.05) had been determined by two‐sided one‐way ANOVA. Asterisks indicate statistically significant differences from the WT control (*P* < 0.05) according to two‐sided Student's *t*‐test.

While no consistent changes in the relative abundance of PsaA, AtpB, and CP47 were observed among genotypes, KO mutant D1 levels were found decreased relative to WT when adjusting for both total protein content (cell homogenate; −27%, *P* = 0.002) or chlorophyll (thylakoid preparation; −39%, *P* = 0.028) (Figure 2f, g). This decrease in D1 coincided with reduced average cellular APC + PC levels detected by in‐gel fluorescence in both crude extracts (−21%; *P* = 0.096) and protein‐adjusted cell homogenate (−35%; *P* = 0.030; Figure 2f, g), thus corroborating previous results indicating phycobilisome depletion in an independent *curT* KO mutant strain (Heinz et al., 2016). The latter was also in line with a marked relative decrease in KO whole‐cell absorbance in the range of 630–660 nm (Figure 3b) and previous reports of PC depletion following mutational loss of PSII in *Synechocystis* (Kılıç et al., 2022).

The observed relative depletion of D1 in absence of equal depletion of CP47 likely points toward a PSII assembly or repair defect in the *curT* KO mutant rather than an overall reduction in cellular PSII complex levels. To scrutinize whether cellular PSI:PSII ratios are affected in the KO strain, low‐temperature (77 K) fluorescence spectrometry was performed. Fluorescence emission spectra excited at 438 nm (Chl *a*) showed peaks at 660 nm (allophycocyanin, APC), 685 nm and 695 nm (PSII with CP43 and CP47), and 720 nm (PSI) (Akhtar et al., 2022; Andrizhiyevskaya et al., 2005; van Stokkum et al., 2023; Figure S3a). The KO mutant displayed increased fluorescence at 660 nm (F660) and 682 nm (F682) relative to the WT, the latter being consistent with previous reports based on an independent, photomixotrophically grown *curT* KO mutant (Heinz et al., 2016; Figure S3a). Reduced excitation energy transfer from the phycobilisomes to PSII may account for APC emission (F660), suggesting partial phycobilisome uncoupling (Remelli & Santabarbara, 2018). This is especially pronounced in the KO mutant (Figure S3a) but was also observed to a lesser degree in the other strains. Similarly pronounced F660 emission under chlorophyll excitation has previously been observed in *Synechocystis* WT samples (Cunningham Jr et al., 2010; Wang et al., 2013), but its precise mechanistic basis remains unresolved. Compromised excitation energy transfer from phycobilisomes to PSII in the *curT* KO strain was further substantiated by emission spectra recorded upon excitation of phycobilisomes at 600 nm, revealing a stark relative increase in F660 in the KO strain as compared to WT (Figure S3c), again pointing toward phycobilisome decoupling (Chukhutsina et al., 2015). Meanwhile, the KO mutant uniquely showed increased relative F682 (Figure S3a,b). While an increase in F685 was previously attributed to increased IsiA levels in a *curT* KO mutant (Heinz et al., 2016) and may overlap with the observed F682 signal, a specific increase in F682 has previously been documented in Mn^2+^‐deficient *Synechocystis* cells and was interpreted as evidence of a PSII assembly defect (Lamb & Hohmann‐Marriott, 2017; Salomon & Keren, 2011). If Mn^2+^ delivery to nascent PSII preferentially occurs in the PratA‐defined membrane domains associated with TCZs as previously hypothesized (Stengel et al., 2012), increased F682 specific to the KO mutant may point toward restored TCZ formation in the compl. mutant strain being sufficient to rescue the underlying PSII defect. A PSII donor‐side defect of the CurT KO mutant is also in line with indication of inefficient phycobilisome binding in Mn_4_Ca‐complex‐deficient D1‐D170A *Synechocystis* mutants (Hwang et al., 2008), again in line with observed indication of phycobilisome uncoupling (Figure S3). Meanwhile, relative emission peaks for PSI (F725) and PSII (F695; CP47) appear largely unchanged, indicating cellular PSI‐to‐PSII ratios to remain stable in the CurT expression mutant strains under photoautotrophic conditions and, together with immunoblot data, pointing toward a symmetrical reduction in PSI and PSII in the KO mutant.

CurT has been crucially implicated in TCZ formation (Heinz et al., 2016), and TCZs together with adjacent PM regions (PratA‐defined membranes, PDMs) have been proposed as PSII biogenesis centers where, among others, Mn^2+^ delivery into nascent PSII is hypothesized to take place (Ostermeier et al., 2025; Stengel et al., 2012). To assess the physiological effects of altered CurT abundance on PSII function beyond immunoblot and 77 K fluorescence analyses, PSII performance parameters (*in vivo* fluorescence, physiological net‐oxygen evolution, enzymatic activity) and cellular pigment composition were investigated. Whole‐cell absorbance spectra (Figure 3a) and methanolic pigment extracts (Figure 3c,d) revealed that CurT depletion significantly increased abundance of cellular carotenoids (Car) relative to chlorophyll *a* (Chl *a*) in both KO (+62%, *P* < 0.001) and compl. strains (+6%, *P* = 0.022), but not OE (−1%, *P* = 0.90; Figure 3c,d). Absolute Chl *a* content per unit OD_750_ was found unaltered in OE and compl., but decreased in KO (−18%; *P* = 0.002) as compared to WT (Figure 3c), corroborating previous observations from an independent *curT* deletion mutant (Heinz et al., 2016). Together this indicates that under photoautotrophic conditions depletion of cellular CurT and/or downstream effects thereof increase cellular carotenoid accumulation.

PSII performance assays indicated impaired photochemistry in CurT‐depleted strains, whereas OE cells performed slightly better than WT regarding both quantum yield estimates and net‐oxygen evolution per unit OD_750_. Specifically, PSII maximum quantum yield QY_max_ was reduced in both KO (−17%, *P* = 0.012) and compl. (−13%, *P =* 0.086), while OE cells showed a statistically insignificant increase (+8%, *P* = 0.310) as compared to WT (Figure 3e). A similar trend was observed under low‐light intensities in light curves of apparent QY measured in absence of DCMU (Figure 3f), while high‐light conditions above 320 μmol photons m^−2^ s^−1^ induced convergence of apparent light‐adapted QY estimates. Despite physiological estimates of PSII quantum yield in cyanobacteria being quantitatively compromised in absence of herbicides (Ogawa et al., 2017), these results indicate a detrimental effect of CurT depletion or the differential loss of CurT‐mediated thylakoid structural features on PSII functionality, which is compatible with a previously proposed functionality of TCZs in PSII biogenesis (Ostermeier et al., 2025, Stengel et al., 2012). Meanwhile, even modest increases of QY in OE especially under low‐light may account for enhanced late‐stage culture cell density (Figure 2a,b). Meanwhile, the decline in light‐adapted quantum yield estimates of compl. mutant cells beyond those of fully segregated *curT* KO mutants (Figure 2e) is puzzling. This effect might be linked to differential effects of complete *versus* partial CurT depletion on phycobilisome coupling in the KO and compl. mutants (Figure S3), but might also hint at compensatory second‐site mutations which have been shown to emerge in *curT* KO strains (Ostermeier et al., 2022) and which might be absent from compl. mutant strains. To rule out that the *curT* KO phenotype originates from such second‐site mutations, two additional independent *curT* KO mutants were generated as reference material, showing the same pigmentation and PSII fluorescence phenotype as the original *curT* KO strain (Figure S4), thus indicating general robustness of the here‐described physiological effects of CurT depletion on PSII and cellular pigmentation. Still, the precise origin of the depressed light‐adapted apparent PSII quantum yield in the compl. strain requires further investigation. Finally, PSII enzymatic activity assessed as steady‐state oxygen evolution rate in presence of 0.5 mM DCBQ and 1 mM potassium ferricyanide was found markedly reduced in both KO and compl. strains on a per‐cell (KO −54%, compl. −22%; Figure 3h) and per Chl *a* content (KO −81%, compl. −32%; Figure 3i). WT PSII activity closely matched independent estimates obtained from WT material derived from the same *Synechocystis* sp. PCC 6803 lineage (Figueroa‐Gonzalez et al., 2026), supporting measurement accuracy and corroborating the implications of both quantum yield and net‐oxygen evolution data regarding CurT depletion‐related impairment of PSII.

As both *Synechocystis* mutants with critically destabilized oxygen‐evolving complex (Δ*psbO* and Δ*psbV*) and D1‐processing mutants (D1‐S345P and Δ*ctpA*) have previously been shown to exhibit reductions in PSII activity (2–5% residual PSII activity per μg Chl *a*; Komenda et al., 2010) far beyond those observed in the here‐examined CurT‐depleted mutants (20–70% of WT levels in *curT* KO and compl. mutants; Figure 3i), any CurT depletion‐related PSII defect implied by our data has to be considered relatively modest. While low‐temperature fluorescence data may point toward a moderate Mn^2+^‐related assembly defect specific to the KO mutant strain (Figure S3), no such indication has been observed to explain reduced PSII activity in the compl. strain. Therefore, an additional CurT‐related PSII assembly or repair defect may account for reduced oxygen evolution capacity in both the KO and compl. strain (Figure 3h,i), which is in line with fast fluorescence (OJIP) transients in presence of DCMU showing a delayed fluorescence increase specifically in the KO, but not in the compl. strain (Figure 3j). This is in line with delayed P‐level attainment in the KO mutant in absence of DCMU (Figure S4d) (Holzer et al., 2026) and compatible with a PSII donor‐side limitation causing enhanced Q_A_^−^ charge recombination (Vass et al., 1999) but could also result from reduced light harvesting.

Collectively, the results presented above indicate that cellular CurT levels affect D1 and phycobiliprotein antenna accumulation (Figures 2f,g and 3a,b), as well as photochemical performance under photoautotrophic conditions (Figure 3e–i), especially in the KO mutant strain. CurT depletion appears to result in compromised D1 abundance (Figure 2f,g), elevates cellular carotenoid content (Figure 3c,d), slows growth (Figure 2a,b), and lowers PSII light‐adapted quantum yield, net‐oxygen evolution, and PSII enzymatic activity (Figure 3e–i). An increased F682 signal specifically observed in KO mutant 77 K emission spectra moreover points toward an Mn^2+^‐related PSII assembly defect (Figure S3), while reduced PSII activity in absence of an F682‐increase in CurT‐depleted compl. mutants may point toward an additional Mn^2+^‐independent assembly or repair defect in PSII. This data underpins a dosage‐dependent role of CurT or CurT‐mediated thylakoid structures on PSII assembly in *Synechocystis*. As significantly lower per‐cell PSII activity in the compl. strain does not translate into a significant growth defect, PSII activity does not appear to be the primary limiting factor for cellular growth of *Synechocystis* under the conditions assayed in this study (i.e., under photoautotrophic growth in BG11 media at 25 °C and 50 μmol photons m^−2^ s^−1^ PAR). In contrast, CurT overexpression modestly enhances net‐oxygen evolution and late‐stage culture density, but may even slightly disrupt PSII accumulation (Figure 2f,g) and PSII activity (Figure 3h,i). This indicates that tight CurT homeostasis and CurT‐related thylakoid structural features are likely critical for balanced PSII biogenesis and effective photoprotection.

### Correlation analysis

**Figure 4:**
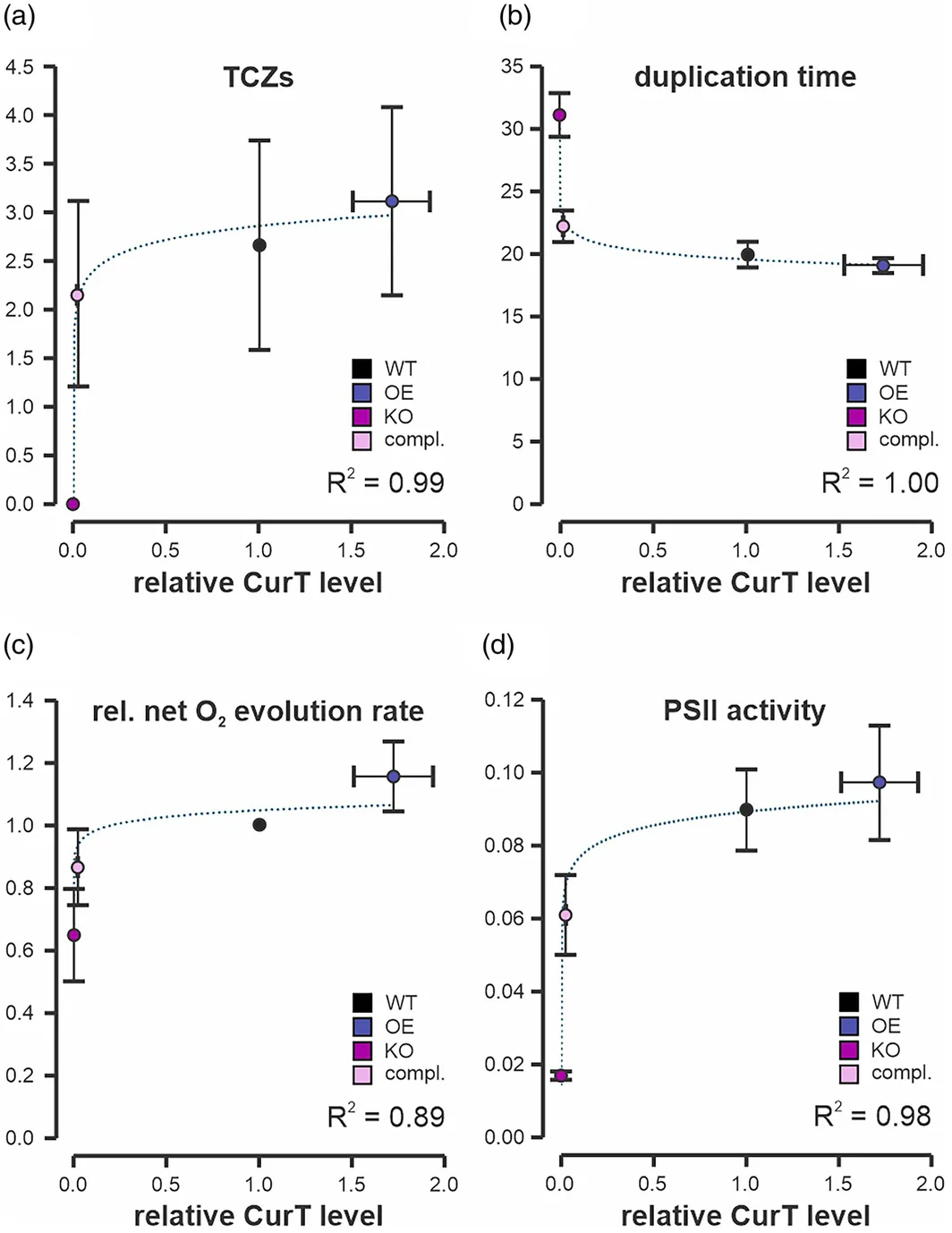
Thylakoid structural features, cell division rates, and PSII activity are a logarithmic function of *Synechocystis* cellular CurT protein levels. Cellular CurT protein levels as determined by whole‐cell crude protein extract immunoblot analysis (Figure 2f; Source Data) were correlated with (a) the number of thylakoid convergence zones (TCZs) per thin section of mid‐exponential growth phase *Synechocystis curT* expression‐level mutant cells (Figure 4c; logarithmic trend function: y = 0.2073ln(x) + 2.8781), (b) duplication times of mid‐exponential growth phase *Synechocystis curT* expression‐level mutant cells (Figure 2b; logarithmic trend function: y = −0.834ln(x) + 19.424), (c) net‐oxygen (O_2_) evolution rates per unit OD_750_ relative to WT measured in light‐adapted mid‐exponential growth phase *Synechocystis curT* expression‐level mutants at 40 μmol photons m^−2^ s^−1^ and 25 °C, closely approximating cultivation conditions (Figure 3g; logarithmic trend function: y = 0.0309ln(x) + 1.0462), and (d) PSII enzymatic activity in units of nmol O_2_ s^−1^ μg Chl *a* ^−1^ (Figure 3i; logarithmic trend function: y = 0.0045ln(x) + 0.0744). Colored circles indicate average values, and error bars indicate standard deviations (vertical: TCZs/duplication time/relative net O_2_ evolution rate/PSII activity; *n* = 3–4) or standard errors (horizontal: relative cellular CurT levels; *n* = 8/11 for OE/compl., respectively. Coefficients of determination R^2^ for logarithmic trends (dotted lines) are provided within each graph.

To quantify the relationship between cellular CurT, thylakoid architecture, and photosynthetic performance, we correlated relative CurT protein levels (Figure 2g; additional crude whole‐cell protein extract immunoblot data provided in Source Data) with structural and physiological parameters using logarithmic regression models (Figure 4). Close logarithmic correlation with cellular CurT levels was observed for abundance of cellular TCZs (R^2^ = 0.99) and exponential growth phase cell duplication time (R^2^ = 1.00) (Figure 4a,b), underpinning CurT as causal agent of TCZ formation (Heinz et al., 2016) and matching previous observations of low levels of cellular CurT facilitating efficient photomixotrophic growth (Dann et al., 2025). Likewise, close logarithmic correlations were observed for cellular CurT and net‐oxygen evolution rates under low‐light conditions approximating the cultivation regime (i.e., 40 μmol photon m^−2^ s^−1^; R^2^ = 0.89) and PSII enzymatic activity (R^2^ = 0.98) (Figure 4c,d). In all cases, correlated ultrastructural and physiological parameters were characterized by steep increases upon injection of trace amounts of CurT into the system. Importantly, these findings indicate that CurT functions as a low‐dosage architectural determinant of thylakoid organization and a facilitator of cellular growth but argue against its large‐scale structural involvement in shaping thylakoid architecture given the largely WT‐like morphology of compl. strain thylakoids (Figure 1a). Instead, other yet‐to‐be identified protein factors may be involved, or CurT‐dependent recruitment of non‐bilayer‐forming galactolipids (Böde et al., 2025; Murphy, 1982) or induced changes in lipid density (de Jesus et al., 2013) may play a crucial role in promoting and stabilizing localized TM curvature, which may in turn mediate the recruitment of other specific protein components (Vanni et al., 2014). Meanwhile, increases in cellular CurT levels enhance both, number of thylakoid layers per peripheral fascicles (Figure 1b) and cellular growth (Figure 2a), albeit at diminishing returns as indicated by the flat plateau phases common to all four logarithmic regressions (Figure 4). WT CurT abundance therefore likely represents a physiological optimum under natural growth conditions, while artificial elevation of CurT levels may improve biomass yield in controlled culture systems as indicated by increased late‐culture OD and robust OD‐to‐dry‐mass correlation (Figure 2c,d), presumably through reduced intracellular self‐shading, and thus suggesting potential for biotechnological application.

### CurT‐related thylakoid ultrastructure modification persists under low‐light

Reduced self‐shading through mutational truncation of peripheral antenna has previously been shown to enhance growth in both plants (Kirst et al., 2017) and cyanobacteria (Sengupta et al., 2023), but to our knowledge, no such effect has yet been reported to result from changes in thylakoid architecture *per se* as indicated for CurT OE strains. Here, additional protruding thylakoid lamellae may facilitate light harvesting in CurT‐over‐accumulating cells, in line with correlative light and electron microscopy (CELM) data confirming the photosynthetic activity of such internal thylakoid membranes (Lübben et al., 2024; Ostermeier et al., 2022). A slight enhancement of such light harvesting capacity may therefore be the cause of minor increases observed in late‐stage culture density, QY_max_, and QY at low‐light intensity (20 μmol photons m^−2^ s^−1^) in OE mutant strains (Figures 2c and 3e,f). As cyanobacteria respond to changes in environmental light intensity by altering both structure and abundance of their thylakoids (Huokko et al., 2021; Liberton et al., 2013), the robustness of CurT dosage‐related thylakoid remodeling remained to be investigated.

**Figure 5:**
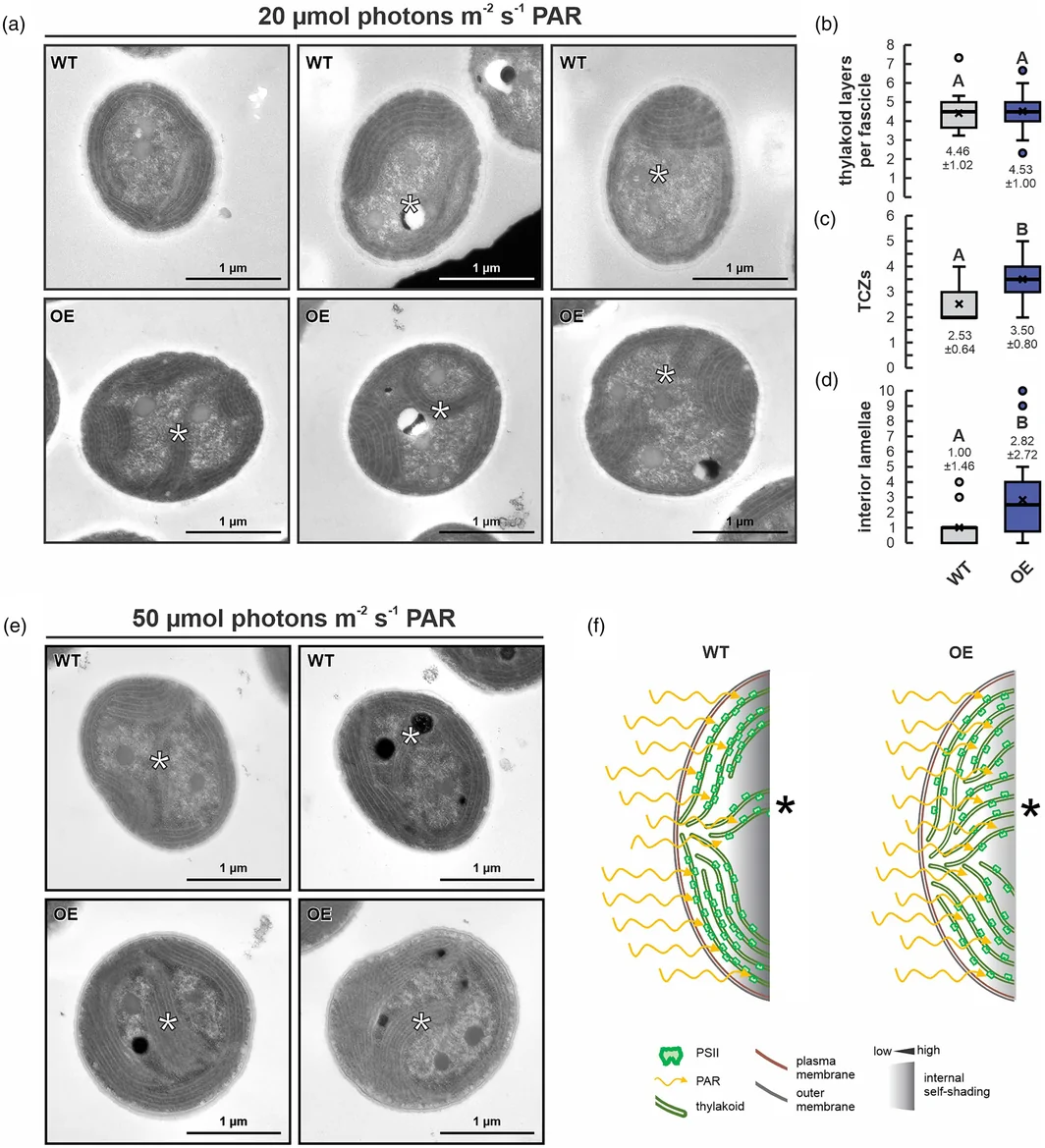
Structural basis for enhanced PSII performance parameters in *Synechocystis curT* overexpression mutants despite low cellular PSII. (a) Representative thin sections transmission electron micrographs of *Synechocystis* WT and *curT* OE mutant cells grown under 20 μmol photons m^−2^ s^−1^ PAR illustrate increased numbers of thylakoid lamellae protruding into the center of OE cells (white asteriks). Scale bars 1 μm. (b) Average number of thylakoid layers per peripheral fascicle (*i.e*., thylakoid‐system segments interrupted by TCZs, see white asterisks in (a, c)) in mid‐exponential growth phase *Synechocystis* WT and *curT* OE mutant cells. (c) Number of thylakoid convergence zones in mid‐exponential growth phase WT and *curT* OE mutant cells. (d) Number of thylakoid lamellae protruding into the cell center of mid‐exponential growth phase WT and *curT* OE mutant cells. (e) Representative thin sections transmission electron micrographs of *Synechocystis* WT and *curT* OE mutant cells grown under 50 μmol photons m^−2^ s^−1^ PAR illustrate increased numbers of thylakoid lamellae protruding into the center of OE cells (see also Figure 1d). Scale bars (1 μm) are indicated. Asterisks indicate interior thylakoid lamellae. (f) Schematic representation of thylakoid structure and PSII distribution throughout the cell in wildtype (WT) and *curT* overexpression (OE) mutant cells. Comparably high density of PSII in the cell periphery presumably leads to self‐shading under low‐light conditions in WT, while PSII‐depleted thylakoid convergence zones (Heinz et al., 2016) reduce local photon absorption efficiency. Additional thylakoid layers and lamellae protruding into the cell center may counteract these shortcomings in OE cells, resulting in lower specific PSII density per unit area of thylakoid and an overall more even PSII distribution throughout the cell, thus possibly improving photon absorption and utilization. PAR, photosynthetically active radiation. Asterisks indicate interior thylakoid lamellae. In panels b–d, boxplots represent data of *n* = 15/22 individual cells for WT/OE, respectively. Estimates are derived from one thin section per observed cell. Boxplots center lines indicate medians, crosses indicate averages, boxes indicate the 25th–75th percentiles, whiskers indicate the 1.5‐fold interquartile range, and circles indicate outliers. Values below plots indicate averages ± standard deviation. Uppercase letters indicate statistically significant differences (*P* < 0.05) according to two‐sided *t*‐test.

To assess whether changes in thylakoid ultrastructure of CurT OE mutant cells persist under constant low‐light conditions and thus may be employed to foster low‐light photosynthesis, cells grown photoautotrophically under low light (20 μmol photons m^−2^ s^−1^ PAR) were investigated by TEM. Low‐light‐grown cells displayed similar ultrastructural changes as observed under standard light intensity (50 μmol photons m^−2^ s^−1^ PAR), with OE cells showing a significant increase in average number of TCZs (+38%; *P* < 0.001) and interior thylakoid lamellae (+180%; *P* = 0.013) as compared to WT (Figure 5a–d), indicating a stable quantitative effect on thylakoid structure due to *curT* overexpression under reduced photon flux density. Meanwhile, under elevated photon flux density (300 μmol photons m^−2^ s^−1^ PAR) shown to induce convergence of quantum yield and net‐oxygen evolution in OE and WT (Figure 3e–g), no discernible differences were observed between WT and OE thylakoid structure (Figure S5). Under elevated light, both WT and OE accumulated a high amount of low‐electron‐density granules between the TMs, possibly corresponding to glycogen built as a result of high photosynthetic activity. Large accumulations of this substance commonly overlapped with tentative TCZs, thus distorting the TEM signal and obstructing quantitative analysis. Meanwhile, CurT protein levels were found unaltered in WT cells grown under both low (109 ± 17%; *P* = 0.594) and elevated light (118 ± 22%; *P* = 0.895) as compared to control light (Figure S6). This indicates per‐cell CurT accumulation to be largely irresponsive to fluctuations in light intensity within the assayed range of 20–300 μmol photons m^−2^ s^−1^ PAR in the WT, and is in line with previous *Synechocystis* proteomics studies that did not find CurT (Slr0483) to be differentially expressed under 300 μmol photons m^−2^ s^−1^ PAR high‐light conditions (Hong et al., 2014).

In summary, our data shows that overexpression of CurT can modify the thylakoid ultrastructure of *Synechocystis* in a predictable manner under low light (Figure 5a–d), thus possibly enabling the tailoring of production strains toward enhanced low‐light or late‐culture productivity through reduced intracellular self‐shading (Figure 5f), while differences in thylakoid structure and the relative fitness effects of CurT depletion are alleviated under elevated and high‐light intensities.

## CONCLUSION

Our results establish CurT as a dosage‐dependent key determinant of thylakoid architecture and promoter of PSII performance in *Synechocystis*, and that both loss and induced overabundance of CurT‐induced ultrastructural features coincide with largely diametrical physiological outcomes. Controlled modulation of cellular CurT levels produces predictable changes in thylakoid sheet number and TCZ abundance and correlates tightly with growth and net‐oxygen evolution. Low performance of CurT‐depleted cells appears to stem from impaired PSII activity compatible with PSII assembly defects, especially in the KO strain, while enhanced photosynthetic productivity in OE appears to stem from thylakoid ultrastructure‐related improvements in light‐energy capture rather than enhanced specific activity of PSII. With recent evidence pointing toward partial conservation of CurT functionality in thylakoid and PSII biogenesis (Zhang et al., 2025) and previous reports of thylakoid lamellae extending toward the plasma membrane during early thylakoid biogenesis in thylapse‐like structures (Huokko et al., 2021) in *Synechococcus elongatus* PCC 7942, broader applicability of our findings to other cyanobacteria lacking typical TCZs appears plausible.

Importantly, this study indicates that TCZs are not merely structural features but have physiological significance: CurT depletion‐related TCZ reduction correlates with low PSII activity, spectral evidence for assembly defects akin to effects of Mn^2+^ limitation, low net O_2_‐evolution, and delayed growth. This is compatible with a model of TCZs acting – non‐exclusively yet preferentially – as microdomains that concentrate PSII assembly factors and intermediates, while physically shortening diffusion pathways between assembly‐factor enriched domains and damaged PSII in the thylakoid periphery, thereby enhancing photosynthetic performance. Meanwhile, largely WT‐like growth of the CurT‐depleted *curT* compl. mutant under control light and of the *curT* KO mutant under high light indicates that under both conditions enough functional PSII is maintained to support growth, compatible with uncompromised PSII repair in absence of sufficient amounts of cellular CurT.

## MATERIALS AND METHODS

### Cyanobacterial strains, culture conditions, and growth curve collection

Experiments were conducted with glucose‐tolerant *Synechocystis* sp. PCC 6803 strains as used in (Dann et al., 2025). The mutant set comprised *curT* knock‐out mutants with the *curT* coding sequence (ORF slr0483) replaced by a spectinomycin resistance marker (KO), overexpression mutants with an additional *curT* gene copy inserted into the genomic neutral site slr0168 (OE), and complementation strains carrying both the KO and OE mutant alleles. All mutant alleles were shown to be fully segregated (Dann et al., 2025). For maintenance, cultures were routinely grown under continuous illumination with 40 μmol photons m^−2^ s^−1^ white fluorescent light (shaker cultures: 5000 K, FHF32EX‐N‐HX‐S; plate cultures: 4000 K; MonotaRO Co. Ltd., Hyogo, Japan) at 25 °C. For growth on solid media, BG11 was supplemented with 0.75% (w/v) bacteriological agar, 1 mM TES/KOH (pH 8.4), and 4 g L^−1^ sodium thiosulfate. Pre‐cultures were grown with suitable antibiotics added to the culture medium (KO, OE: 25 μg ml^−1^ spectinomycin; compl.: 25 μg ml^−1^ spectinomycin + 8.5 μg ml^−1^ chloramphenicol), while assay cultures were grown without antibiotics to minimize physiological stress. Assay liquid cultures were inoculated at OD_730=_0.05 in BG11 medium without added glucose (Rippka et al., 1979) and cultivated in a Multi‐Cultivator MC 1000‐OD (Photon Systems Instruments, Drásov, Czech Republic) under 50 μmol photons m^−2^ s^−1^ of warm‐white LED light and atmospheric aeration, collecting growth curves in 60‐min intervals using the built‐in MC 1000‐OD photometer. In the late exponential and early plateau phase, corresponding OD_720_ values collected from undiluted cultures represent underestimates of the ‘true’ OD_720_ as measured in conventional 10 mm cuvettes due to the lack of dilution and thickness of the culture layer assayed (27 mm diameter of culture vessels), which is why these values are labeled ‘apparent OD_720_’. Assay culture samples for biochemical, physiological, and microscopic analyses were collected within the exponential growth phase, and total cultivation time approximately 150–160 h. For low‐ and high‐light experiments, cells were grown under 20 and 300 μmol photons m^−2^ s^−1^, respectively, under otherwise identical conditions. *Synechocystis* strains were grown at 25 °C in order to preserve direct comparability with previous studies on the same mutant material (Dann et al., 2025).

*Synechocystis* duplication time estimates were calculated after the exponential growth phase was determined on a logarithmic scale plot for all strains as t_d_ = LN(2)/([LN(OD_t1_)/LN(OD_t0_)]/[_t1_‐_t0_]). Exponential growth phases were observed to occur between 29 (t_0_) and 56 (t_1_) hours past inoculation for WT, OE, and compl., and between 40 (t_0_) and 64 (t_1_) hours past inoculation for KO mutant strains.

### *Synechocystis* dry‐weight determination

*Synechocystis* exponential growth phase culture dry weight was determined by harvesting cell material from 15 ml culture volume by centrifugation (10 min, 3200 × ***g***), washing cells with 1 ml of BG11 media, and subsequently drying cell pellets completely in a rotary evaporator. Dry weight was then related to original OD_750_ determined for the corresponding cultures.

### Determination of *Synechocystis* cell count per unit OD_730_

To determine the cell count per unit OD_730_ in *Synechocystis curT* expression‐level mutants, cells of exponential growth phase cultures were harvested and adjusted to OD_730_ = 0.8 and subsequently counted in a Neubauer‐improved counting chamber (Carl Roth, Karlsruhe, Germany) under a light microscope ZEISS Axioskop (ZEISS, Oberkochen, Germany).

### Protein extraction, detection, and quantification

*Synechocystis* crude whole‐cell protein extracts were prepared as described by (Dann et al., 2021) from cells grown in liquid culture for approximately 3 days to mid‐exponential growth phase. Culture samples were normalized to OD_750_, harvested by centrifugation, and cells were dissolved in lysis buffer (Tris 100 mM – pH 6.8 (HCl); glycerol 24% (v/v); SDS 5% (w/v); bromophenol blue 0.02% (w/v); DTT 100 mM) at 95 °C for 5 min under vigorous agitation (1200 rpm). Equivalents of OD_750_ = 4.0 cells were lysed in 100 μl lysis buffer and debris was excluded by centrifugation prior to loading. *Synechocystis* whole‐cell homogenate and thylakoid membranes were prepared as previously described (Gandini et al., 2017). In brief, *Synechocystis* cells from 50 mL mid‐exponential growth phase culture volume were harvested by centrifugation and resuspended in 1 mL of homogenization buffer (0.4 M sucrose, 10 mM NaCl, 5 mM MgCl_2_, 20 mM Tricine, pH 7.9, 10 mM Na‐ascorbate, 10 mM NaF). Cells were then ruptured using a bead mill homogenizer and a mix of glass beads (0.25–1.0 mm) at 4 °C and 30 Hz in the dark. Thylakoids were collected from whole‐cell homogenate by centrifugation and repeated washing in 5 mM Tricine, 10 mM NaF at 4 °C. Sample concentration was then adjusted to total protein (whole‐cell homogenate) and Chl *a* content (thylakoids). For standard SDS PAGE, 5 μl of crude whole‐cell protein extract sample corresponding to OD_750_ = 0.2 cell equivalents, whole‐cell homogenate corresponding to 30 μg of total protein as determined by Bradford protein assay, or thylakoid preparation corresponding to 5 μg Chl were loaded in 1x lysis buffer. Whole‐cell protein extract corresponding to 30 μg total protein or 5 μg Chl *a* equivalent thylakoid protein samples were fractionated by SDS PAGE on 4% /12% polyacrylamide Tris‐Tricine gels (Schägger & Von Jagow, 1987). Proteins were blotted onto PVDF membrane (Millipore Immobilon‐P Transfer Membrane, pore size 0.45 μm) at 0.1 A, 25 V for 90 min (Trans‐Blot® Turbo™ Transfer System, Bio‐Rad Laboratories, Inc., Hercules, CA, USA), and the CurT protein was immuno‐detected using primary antibody serum raised against the N‐terminus of *Synechocystis* CurT (Armbruster et al., 2013) provided by Dario Leister (LMU Munich) at a 1:10000 dilution. PsbA/D1, PsbB/CP47, AtpB, and PsaA proteins were immuno‐detected using polyclonal primary antibody AS05 084 (D1), AS04 038 (CP47), AS05 085–10 (AtpB), and AS06 172 (PsaA) (Agrisera, Vännäs, Sweden). Combined allophycocyanin and phycocyanin levels (APC + PC) were quantified *via* in‐gel fluorescence using a BIO RAD ChemiDoc MP Imaging System (Alexa 546 fluorescence channel) prior to gel blotting. Immunoblot ECL signals were detected using horse‐radish‐peroxidase conjugated Goat anti‐Rabbit IgG AS09 602 (Agrisera) as secondary antibody at 1:10000 dilution. Chemiluminescence and fluorescence signals were quantified using ImageJ (Schneider et al., 2012), and normalized to the intensity of the corresponding wildtype signal on the respective PVDF membrane. Routinely, calibration curves for protein level intrapolation were obtained based on WT sample titration (200/100/50/25/2.5% of standard loading), and protein level estimates for OE, KO, and compl. mutant strains were derived from the best‐fit regression model (R^2=^0.92–1.0) (Figures S2 and S7). For collection of additional CurT level data points used for correlation analyses (Figure 4), additional immunoblots were analyzed (see Source Data). As not all of these blots included a full WT titration series, protein level approximation was achieved through normalization to 100% WT signals on the respective blots.

### Low‐temperature chlorophyll fluorescence spectrometry (77 K)

The 77 K fluorescence emission spectra of *Synechocystis* cells were measured using a HORIBA Fluoromax Plus FL‐1013 (HORIBA Jobin Yvon GmbH, Bensheim, Germany) after chlorophyll excitation at 438 nm and phycobilisome excitation at 600 nm, adapted from (Klinkert et al., 2004). Culture samples were harvested mid‐exponential growth phase and adjusted to OD_750_ = 3.0 prior to measurement.

### Whole‐cell absorbance spectra

High‐quality whole‐cell absorbance spectra (Figure 3a,b) from mid‐exponential growth phase cultures were obtained from cell suspension initially adjusted to an OD_750_ of 1.65 in BG11 media (estimated using a FastGene® Photometer NanoSpec FG‐NP01; NIPPON Genetics Europe GmbH, Düren, Germany). Spectra were collected with a Perkin Elmer Lambda 35 UV/Vis Spectrometer equipped with an integrating sphere (PerkinElmer, Rodgau, Germany). Whole‐cell absorbance spectra for qualitative comparison of mutant phenotypes (Figure S4c) were obtained from mid‐exponential growth phase cultures with cell suspension adjusted to an OD_750_ of 3 prepared in a final volume of 100 μL. The suspension was mixed with an equal volume of PBST (1× PBS buffer containing 0.1% Tween 20) and centrifuged at 3500 × *g* at room temperature. The resulting pellet was resuspended in 100 μL of PBST buffer. Absorbance between 350 nm and 800 nm was measured using the microvolume mode of a FastGene® Photometer NanoSpec (FG‐NP01; NIPPON Genetics Europe GmbH, Düren, Germany) to minimize scatter.

### Methanolic pigment extraction and quantification

Pigments were extracted and quantified according to the method described by (Zavřel et al., 2015). For pigment analysis, pigments from 1 mL of *Synechocystis* culture with an OD_750_ of 0.75 were pelleted at 9680 × ***g*** for 4 min. The pelleted cells were then resuspended in 1 mL of cold methanol, vortexed, and covered with aluminum foil. Pigments were extracted for 4 h in the refrigerator. The solution was centrifuged at 9680 × ***g*** for 10 min, and the supernatant was decanted into a 10 mm polystyrene cuvette (Sarstedt, Nümbrecht, Germany), and the absorption spectrum was measured between 350 nm and 800 nm (FastGene® Photometer NanoSpec (FG‐NP01), NIPPON Genetics EUROPE GmbH, Düren, Germany).

### Fluorescence analysis to estimate maximum and light‐adapted quantum yields

To estimate the performance of PSII in *Synechocystis* cells, fluorescence light curves and OJIP fluorescence transients were obtained using an AquaPen (AquaPen AP110‐C, Photon Systems Instruments, Drásov, Czech Republic). For light curves, measuring pulses were set to 10%, and the saturating light pulses were set to 50%. To approximate PSII quantum yield (QY) under different light intensities, the cells were dark‐adapted for 10 min before initiating the predefined light curve three (LC3; light intensities in μmol photons m^−2^ s^−1^: 0, 10, 20, 50, 100, 300, 500, 1000). As QY was measured in the absence of DCMU (3‐(3,4‐dichlorophenyl)‐1,1‐dimethylurea), allowing for accurate measurement of maximum fluorescence F_m_, QY estimates are labeled as ‘apparent’. To assess true PSII maximum QY (QY_max_), 10 μM DCMU was added to cell suspensions prior to dark incubation and OJIP transient measurements. The OJIP data were analyzed using the tool https://www.cyano.tools/OJIP_data_analysis.

### O_2_ evolution measurements

Net‐oxygen evolution rate measurements of *Synechocystis* cultures were performed in the absence of added electron acceptor as previously described (Dann et al., 2021). To analyze O_2_ evolution at PSII, ~ 2 ml of cell culture was filled into the chamber of a Clark‐Type Electrode Oxytherm+P (Hansatech Instruments Ltd., Pentney, Norfolk, United Kingdom). Measurements were conducted at 25 °C with light intensity changing every 5 min. The seven light intensity steps in μmol photons m^−2^ s^−1^ were: 0, 40, 120, 320, 480, 960, 0. Net O_2_ evolution was determined by correcting for the respiration rate obtained after the light regimen, and the data were normalized to the cultures' OD_750_. To compare results among three sets of measurements obtained from separate electrode preparations and calibrations, results of the OE, KO, and compl. strains were normalized to the corresponding WT controls.

To assess PSII enzymatic activity, steady‐state oxygen evolution under saturating light was measured in the presence of 0.5 mM DCBQ (2,6‐dichloro‐1,4‐benzoquinone) and 1 mM ferricyanide (potassium hexacyanoferrate, K_3_[Fe(CN)_6_]) as previously described (Figueroa‐Gonzalez et al., 2026) and subsequently normalized to OD_750_ (cell count) or chlorophyll content.

### Transmission electron microscopy

For *Synechocystis* cells, sample preparation was performed as previously described (Lübben et al., 2024). *Synechocystis* cells were pelleted by centrifugation at 2000 × ***g*** for 15 min. The resulting pellets were gently resuspended in 20 μl of culture medium immediately before cryofixation. Samples were frozen under high pressure (2100 bar) using an EM HPM100 (Leica Microsystems, Wetzlar, Germany) and then stored in liquid nitrogen. Cryofixation was followed by freeze substitution in A.O.U.H. solution at −90 °C (acetone containing 0.2% [w/v] OsO₄, 0.1% [w/v] uranyl acetate, and 9% [v/v] H_2_O) for 42 h, as previously described (Peschke et al., 2013; Walther & Ziegler, 2002), using an EM AFS2 (Leica Microsystems). After substitution, samples were embedded in Epon 812 and polymerized for 16 h at 63 °C. Ultrathin sections of 70 nm (ultra 35°, 3.0 mm, DiATOME) were cut on an Ultracut E ultramicrotome (Leica Microsystems) using diamond knives. Sections were collected on collodion‐coated, 400‐mesh copper grids (Science Services GmbH, Munich, Germany). Prior to imaging, ultrathin sections were post‐stained with lead citrate according to (Reynolds, 1963). Samples were examined in a Zeiss EM 912 transmission electron microscope (Zeiss, Oberkochen, Germany) equipped with an integrated OMEGA energy filter operated in zero‐loss mode at 80 kV. Images were captured at a nominal magnification of 12 500× using a 2 k × 2 k slow‐scan CCD camera (Tröndle Restlichtverstärkersystem, Moorenweis, Germany).

### Statistical analyses

Charts were created using Microsoft Office Excel 365. For boxplots, internal datapoints are indicated, horizontal lines represent the median, crosses represent average values, and boxes indicate the 25th and 75th percentiles. Whiskers extend 1.5‐fold the interquartile range, with outliers being represented as circles beyond the range of the whiskers. For pairwise comparison of averages, two‐sided Student's *t*‐tests were performed in Microsoft Excel. For multiple simultaneous comparisons, statistically significant among‐group differences were tested for by one‐way ANOVA (two‐sided), followed by *post hoc* Tukey HSD (honest significant differences) tests with Bonferroni–Holm *P*‐value correction. Significant differences according to multiple simultaneous *post hoc* comparisons are routinely indicated by uppercase letters denoting the resultant groups of not significantly different (same letter) and significantly different (different letter) samples. Analyses were performed using the one‐way ANOVA with *post hoc* test tool as implemented by Navendu Vasavada (https://astatsa.com/).

## AUTHOR CONTRIBUTIONS

MO and MD designed the research; MO, A‐CP, PR, and MD performed research; MO, A‐CP and MD analyzed data; MD wrote the manuscript with contributions from ACP and MO.

## CONFLICT OF INTEREST STATEMENT

The authors declare no conflicts of interest.

## Supporting information


**Figure S1.** Additional transmission electron micrographs of *Synechocystis curT* expression‐level mutant cells. Representative cells of *n* = 37/38/37/35 individual cells imaged for WT/OE/KO/compl., respectively, are shown. Scale bars indicate 1 μm.
**Figure S2.** Calibration curves for *Synechocystis curT* expression mutant comparative immunoblot signal quantification. Calibration curves derived from immunoblot ECL signals (PsaA, AtpB, CP47, D1, CurT) and in‐gel fluorescence signals prior to blotting (APC + PC) shown in Figure 2f (*n* = 3 biological replicates; #1/2/3). Best‐fitting regression models for correlation of protein extract sample input (x‐axis; WT titration) and signal intensity are provided and used for protein level approximation in *curT* OE, KO, and compl. strain samples. Coefficients of determination (R^2^) are indicated. (a) Crude whole‐cell protein extracts (Figure 2f, left). (b) Protein‐adjusted whole‐cell homogenate (Figure 2f, middle). (c) Chlorophyll‐adjusted thylakoid preparation (5 μg Chl a per lane; Figure 2f, right).
**Figure S3.** Low‐temperature (77 K) fluorescence emission spectra of *Synechocystis curT* expression‐level mutants. (a) Low‐temperature fluorescence emission spectra of *curT* expression‐level mutants excited at λ_exc=_438 nm measured at 77 K. Spectra were normalized to PSI emission (F720). Mean spectra of *n* = 8–9 biological replicates each are shown. (b) Ratio of fluorescence (λ_exc_ = 438 nm) at 682 nm (F682; unassembled PSII, (Salomon & Keren, 2011)) to fluorescence at 695 nm (F695; CP47). Columns indicate averages, error bars indicate standard deviations. Asterisk indicates statistically significant difference from the WT control (*P* < 0.05) according to two‐sided Student's *t*‐test. (c) Low‐temperature fluorescence emission spectra of WT and *curT* KO mutants excited at λ_exc_ = 600 nm measured at 77 K. Spectra were normalized to PSI emission (F720). Mean spectra ± standard deviation are shown for *n* = 3 biological replicates each. (d) Ratio of fluorescence (λ_exc_ = 600 nm) at 660 nm (F660; APC) to F695 (left) and F682 to F695. Columns indicate averages, error bars indicate standard deviations. Asterisk indicates statistically significant difference from the WT control (*P* < 0.05) according to two‐sided Student's *t*‐test.
**Figure S4.** Newly generated *curT* knock‐out mutants show similar cellular pigmentation and PSII fluorescence transients as original KO strain. (a) Genotyping PCR verifies complete replacement of chromosomal *curT* coding sequence (ORF slr0483) by spectinomycin resistance cassette as previously described (Dann et al., 2025). Expected amplicon sizes are 1.49 kilo base pairs [kbp] for the WT allele, and 2.53 kpb for the *curT* KO allele. (b) Immunoblot analysis of CurT accumulation in new and original *curT* KO mutants. PVDF membrane Coomassie Brilliant Blue (C.B.B.) staining provided as loading control. Molecular weight markers are indicated. (c) Average whole cell absorbance spectra normalized to the chlorophyll *a* absorbance maximum at λ = 682 nm. Traces represent data from *n* = 1 (KO mutant strains) and *n =* 3 (WT) biological replicates, respectively. (d) OJIP fluorescence transients of newly generated and original *curT* KO mutant strains as compared to the parental WT. Cells were dark‐treated for 10 min prior to measurement. Traces represent average data from *n* = 2 (KO mutant strains) and *n* = 4 (WT) biological replicates, respectively.
**Figure S5.** Transmission electron micrographs of *Synechocystis* WT and *curT* OE mutant cells grown under elevated light intensity. Representative cells of *n* = 20 individual *Synechocystis* cells imaged for WT/OE grown under elevated light intensity of 300 μmol photons m^−2^ s^−1^, respectively, are shown. White arrowheads show low‐electron‐density granula (possibly glycogen) overlapping with tentative TCZs. Scale bars indicate 1 μm.
**Figure S6.** CurT protein level response to low‐ and high‐light conditions. (a) PsaA, D1, and CurT protein levels as assessed by immunoblot analysis of whole‐cell protein extracts obtained from mid‐exponential‐phase cultures grown under control (CL), low (LL), and elevated light (EL) conditions (50/20/300 μmol photons m^−2^ s^−1^ PAR, respectively) and harvested at 3–4 days past inoculation. Combined allophycocyanin and phycocyanin (APC + PC) levels were assessed by in‐gel fluorescent assay prior to blotting of SDS‐PA gels. Loading was adjusted to OD_750_. PVDF membrane Coomassie Brilliant Blue (CBB) staining served as loading control. Molecular weight markers are indicated. (b) Relative protein quantity estimates (CL 100% serving as reference; see Methods). Data shown represent *n* = 4 biological replicates. Columns show averages; error bars indicate standard deviations. Letters (Roman, Greek, Cyrillic) indicate statistically significant differences (*P* < 0.05) according to *post hoc* Bonferroni–Holm‐corrected Tukey HSD after significant among‐group differences (*P* < 0.05) had been determined by two‐sided one‐way ANOVA.
**Figure S7.** Calibration curves for *Synechocystis* WT control−/low−/high‐light culture immunoblot signal quantification. Calibration curves derived from immunoblot ECL signals (PsaA, D1, CurT) and in‐gel fluorescence signals prior to blotting (APC + PC) shown in Figure S6 (plus two more biological replicates; blots G‐H) indicate partially linear (PsaA, CurT, APC + PC), partially logarithmic (D1) correlation of whole‐cell protein extract sample input (x‐axis; 100% corresponding to OD_750_ = 0.2 cell equivalents) and signal intensity relative to 100% control‐light (CL) sample input (y‐axis). Best‐fitting linear or logarithmic regression models were chosen covering the required range for signal quantification of WT samples grown under low and high light (20 and 300 μmol photons m^−2^ s^−1^ PAR) relative to WT grown under CL conditions (50 μmol photons m^−2^ s^−1^ PAR). Calibration curve equations used for protein level approximation and corresponding coefficients of determination (R^2^) are indicated.

Data S1.

Data S2.

## Data Availability

The data that supports the findings of this study are available in the Supporting Information of this article.
