# Supplementary material for: CurT dosage quantitatively affects thylakoid structure and PSII performance in Synechocystis sp. PCC 6803
Source: Plant J. 2026 Sep 21;127(6):e71132. doi: 10.1111/tpj.71132 (PMC13592050; doi:10.1111/tpj.71132)
Supplement: Supplementary file 1 [file TPJ-127-0-s002.docx]

**Supporting Information**

**Figure S1. Additional transmission electron micrographs of *Synechocystis* *curT* expression level mutant cells.** Representative cells of of *n*=37/38/37/35 individual cells imaged for WT/OE/KO/compl., respectively, are shown. Scale bars indicate 1 µm.

**Figure S2. Calibration curves for *Synechocystis* *curT* expression mutant comparative immunoblot signal quantification.**

Calibration curves derived from immunoblot ECL signals (PsaA, AtpB, CP47, D1, CurT) and in-gel fluorescence signals prior to blotting (APC+PC) shown in **Fig. 2f** (*n*=3 biological replicates; #1/2/3). Best fitting regression models for correlation of protein extract sample input (x-axis; WT titration) and signal intensity are provided and used for protein level approximation in *curT* OE, KO, and compl. strain samples. Coefficients of determination (R^2^) are indicated.

(a) Crude whole-cell protein extracts (Fig. 2f, left).
(b) Protein-adjusted whole-cell homogenate (Fig. 2f, middle).
(c) Chlorophyll-adjusted thylakoid preparation (5 µg Chl a per lane; Fig. 2f, right).

**Figure S3. Low-temperature (77 K) fluorescence emission spectra of *Synechocystis curT* expression level mutants.**

(**a**) Low-temperature fluorescence emission spectra of *curT* expression level mutants excited at λ_exc=_438 nm measured at 77 K. Spectra were normalized to PSI emission (F720). Mean spectra of *n* =8-9 biological replicates each are shown.
(**b**) Ratio of fluorescence (λ_exc_=438 nm) at 682 nm (F682; unassembled PSII, (Salomon and Keren, 2011)) to fluorescence at 695 nm (F695; CP47). Columns indicate averages, error bars indicate standard deviations. Asterisk indicates statistically significant difference from the WT control (*p*<0.05) according to two-sided Student’s *t*-test.
(**c**) Low-temperature fluorescence emission spectra of WT and *curT* KO mutants excited at λ_exc_=600 nm measured at 77 K. Spectra were normalized to PSI emission (F720). Mean spectra ± standard deviation are shown for *n* =3 biological replicates each.
(**d**) Ratio of fluorescence (λ_exc_=600 nm) at 660 nm (F660; APC) to F695 (left) and F682 to F695. Columns indicate averages, error bars indicate standard deviations. Asterisk indicates statistically significant difference from the WT control (*p*<0.05) according to two-sided Student’s *t*-test.

**Figure S4. Newly-generated *curT* knock-out mutants show similar cellular pigmentation and PSII fluorescence transients as original KO strain.**

(**a**) Genotyping PCR verifies complete replacement of chromosomal *curT* coding sequence (ORF slr0483) by spectinomycin resistance cassette as previously described (Dann *et al.*, 2025). Expected amplicon sizes are 1.49 kilo base pairs [kbp] for the WT allele, and 2.53 kpb for the *curT* KO allele.

(**b**) Immunoblot analysis of CurT accumulation in new and original *curT* KO mutants. PVDF membrane Coomassie Brilliant Blue (C.B.B.) staining provided as loading control. Molecular weight markers are indicated.

(**c**) Average whole cell absorbance spectra normalized to the chlorophyll *a* absorbance maximum at λ=682 nm. Traces represent data from *n*=1 (KO mutant strains) and *n=*3 (WT) biological replicates, respectively.

(**d**) OJIP fluorescence transients of newly generated and original *curT* KO mutant strains as compared to the parental WT. Cells were dark-treated for 10 min prior to measurement. Traces represent average data from *n*=2 (KO mutant strains) and *n*=4 (WT) biological replicates, respectively.

**Figure S5. Transmission electron micrographs of *Synechocystis* WT and *curT* OE mutant cells grown under elevated light intensity.**
Representative cells of *n*=20 individual *Synechocystis* cells imaged for WT/OE grown under elevated light intensity of 300 µmol photons m^-2^ s^-1^, respectively, are shown. White arrowheads show low electron density granula (possibly glycogen) overlapping with tentative TCZs. Scale bars indicate 1 µm.

**Figure S6. CurT protein level response to low and high light conditions.**

(**a**) PsaA, D1, and CurT protein levels as assessed by immunoblot analysis of whole-cell protein extracts obtained from mid-exponential phase cultures grown under control (CL), low (LL), and elevated light (EL) conditions (50/20/300 µmol photons m^-2^ s^-1^ PAR, respectively) and harvested at 3-4 days past inoculation. Combined allophycocyanin and phycocyanin (APC+PC) levels were assessed by in-gel fluorescent assay prior to blotting of SDS-PA gels. Loading was adjusted to OD_750_. PVDF membrane Coomassie Brilliant Blue (CBB) staining served as loading control. Molecular weight markers are indicated.

(**b**) Relative protein quantity estimates (CL 100% serving as reference; see Methods). Data shown represents *n*=4 biological replicates. Columns show averages; error bars indicate standard deviations. Letters (Roman, Greek, Cyrillic) indicate statistically significant differences (*p*<0.05) according to *post-hoc* Bonferroni-Holm-corrected Tukey HSD after significant among-group differences (*p*<0.05) had been determined by two-sided one-way ANOVA.

**Figure S7. Calibration curves for *Synechocystis* WT control-/low-/high-light culture immunoblot signal quantification.**

Calibration curves derived from immunoblot ECL signals (PsaA, D1, CurT) and in-gel fluorescence signals prior to blotting (APC+PC) shown in **Fig. S6** (plus two more biological replicates; blots G-H) indicate partially linear (PsaA, CurT, APC+PC), partially logarithmic (D1) correlation of whole-cell protein extract sample input (x-axis; 100% corresponding to OD_750_=0.2 cell equivalents) and signal intensity relative to 100% control-light (CL) sample input (y-axis). Best-fitting linear or logarithmic regression models were chosen covering the required range for signal quantification of WT samples grown under low and high light (20 and 300 µmol photons m^-2^ s^-1^ PAR) relative to WT grown under CL conditions (50 µmol photons m^-2^ s^-1^ PAR). Calibration curve equations used for protein level approximation and corresponding coefficients of determination (R^2^) are indicated.
